# The Reflective Functioning Questionnaire–Revised– 7 (RFQ-R-7): A new measurement model assessing hypomentalization

**DOI:** 10.1371/journal.pone.0282000

**Published:** 2023-02-24

**Authors:** Zsolt Horváth, Orsolya Demetrovics, Borbála Paksi, Zsolt Unoka, Zsolt Demetrovics

**Affiliations:** 1 Institute of Psychology, ELTE Eötvös Loránd University, Budapest, Hungary; 2 Centre of Excellence in Responsible Gaming, University of Gibraltar, Gibraltar, Gibraltar; 3 Centre of Mental Health, Pál Heim Hospital, Budapest, Hungary; 4 Institute of Education, ELTE Eötvös Loránd University, Budapest, Hungary; 5 Department of Psychiatry and Psychotherapy, Semmelweis University, Budapest, Hungary; Universita degli Studi Europea di Roma, ITALY

## Abstract

Although it is a widely used questionnaire, limitations regarding the scoring procedure and the structural validity of the eight-item Reflective Functioning Questionnaire (RFQ-8) were raised. The present study aimed to examine further the latent dimensionality of the RFQ-8 and to examine linear and non-linear associations between mentalization difficulties and maladaptive psychological characteristics. Data from two separate representative samples of young adults (*N* = 3890; females: 51.68%; mean age: 27.06 years [*SD* = 4.76]) and adults (*N* = 1385; females: 53.20%; mean age: 41.77 years [*SD* = 13.08]) were used. In addition to the RFQ-8, standardized questionnaires measured the levels of impulsivity, sensation seeking, rumination, worry and well-being. Confirmatory factor analysis (CFA) was used to test the model fit of competing measurement models. CFA revealed that a revised, seven-item version of the RFQ (RFQ-R-7) with a unidimensional structure showed the most optimal levels of model fit in both samples. Impulsivity, sensation seeking, rumination and worry consistently presented significant, positive, linear associations with general mentalization difficulties in both samples. Significant quadratic associations were also identified, but these relationships closely followed the linear associations between the variables and increased only marginally the explained variance. The supported unidimensional measurement model and the associations between the general mentalization difficulties factor and maladaptive psychological characteristics indicated that the RFQ-R-7 captures a dimension of hypomentalization ranging between low and high levels of uncertainty. Increasing levels of hypomentalization can indicate a risk for less adaptive psychological functioning. Further revisions of the RFQ-8 might be warranted in the future to ensure adequate measurement for hypermentalization.

## 1. Introduction

Mentalization or reflective functioning (used as synonymous terms in the present article) indicates one’s ability”to reflect on internal mental states such as feelings, wishes, goals, and attitudes, with regard to both the self and others” [1]. Mentalization can be conceptualized as a multidimensional construct. It incorporates various forms of reflective functioning, for example, automatic or controlled forms, self-directed or externally-oriented forms, and cognitive or affective forms [2]. Multiple sociocultural factors can influence the development of reflective functioning and the circularly interrelated capacities of epistemic trust, social learning and secure attachment: parental reflective functioning in attachment context as well as family and neighborhood factors, sociocultural context [2].

Mentalization difficulties, as transdiagnostic risk factors, are associated with numerous psychopathological disorders and symptoms [3], such as borderline and antisocial personality disorders, lower levels of personality organization, major depressive and anxiety disorders (e.g., panic and posttraumatic stress disorders), eating disorders, schizophrenia, autism spectrum disorder [2,4–6]. In line with this, mentalization based treatment approaches are frequently used in mental health care [7–9].

Moreover, impairments in reflective functioning are also associated with other transdiagnostic factors for psychiatric disorders. For example, difficulties in emotion regulation and mentalizing are overlapping constructs to some degree (e.g., the ability to understand emotions is considered in both emotion regulation and mentalization processes) and show neurobiological-level functional relationships. Therefore, previous studies consistently showed positive associations between difficulties in emotion regulation, maladaptive emotion regulation strategies and impairments in reflective functioning in clinical and non-clinical samples [2,10–14]. In addition to this, some studies also reported positive correlations between impulsivity and mentalization difficulties which also can be explained by the similarities between these constructs (e.g., negative urgency-related characteristics, lack of premeditation can be considered as a form of reflective functioning difficulties) [10,11,15]. However, the relationship between other transdiagnostic externalizing personality dimensions, such as sensation seeking, and reflective functioning difficulties was examined limitedly so far. It can be assumed that there might be a positive link between sensation seeking and reflective functioning difficulties also since sensation seeking may also be associated with emotion regulation difficulties, specifically with problems in regulating positive emotions (e.g., reward sensitivity, positive urgency) [16]. Thus, it might even be possible that those with heightened sensation seeking may show reduced mentalizing capacities while experiencing positive emotional states. Moreover, both constructs may share similarities at behaviour level in terms of high disinhibition and lack of premeditation [17].

One of the most widely used self-report questionnaire for measuring mentalization difficulties is the Reflective Functioning Questionnaire (RFQ) [1]. Its brief version contains 8 items (RFQ-8) which aims to measure two distinct types of mentalization difficulties: hypomentalization and hypermentalization. Hypomentalization (or concrete or psychic equivalent thinking) refers to the inability of forming complex models of one’s own and others’ mind, while hypermentalization (i.e., pseudo-mentalizing or excessive mentalizing) is described as forming mentalistic representations about one’s and others’ actions or thoughts and feelings without anchoring them in reality. In other words, hypo- and hypermentalization refers to the too low and too high levels of certainty regarding the interpretation and understanding of internal mental states [1]. For example, hypomentalization may contribute to someone shouting in the company of a young child, as this person may experience difficulty in assessing and taking others’ perspectives on how his or her behavior will affect those around him or her (e.g., shouting may frighten the child). In turn, hypermentalization can contribute, for example, to a person making unrealistic inferences about his or her partner’s intentions (e.g., betrayal, rejection by the other) in response to the partner’s non-immediate response to his or her text message [17]. On the contrary genuine mentalizing refers to the opaqueness of the mental states of both the self and others (i.e., being aware of not having complete understanding and comprehension of mental states), and therefore contains a moderate level of uncertainty. Genuine mentalizing is thus characterized by both some extent of certainty and uncertainty while individuals consider having a decent knowledge about their mental states [1].

Individuals in the RFQ-8 provide responses for each item on a seven-point scale between “strongly disagree” and “strongly agree” response options. However, in order to calculate the subscale scores, the items of the RFQ must be recoded. For example, it was assumed that on the item of “I don’t always know why I do what I do” low scores (response options 1, 2 and 3) indicate hypermentalization (certainty), response option in the middle (response option 4) implicates genuine reflective functioning, whereas high scores indicate hypomentalization (uncertainty, response options 5, 6 and 7). That is, only high and/or low scores on a particular item are considered to calculate each subscale score, and responses on other categories are weighted by 0 point (e.g., the scoring procedure of the aforementioned item for hypermentalization [certainty] subscale: 3, 2, 1, 0, 0, 0, 0; for hypomentalization [uncertainty] subscale: 0, 0, 0, 0, 1, 2, 3). Overall, 6–6 variables form the two subscales and responses on four items of the RFQ-8 are simultaneously considered on both subscales. By using this scoring procedure, various previous studies confirmed the two-factor structure of the RFQ-8 in clinical and non-clinical samples [1,18–21].

However, recent studies highlighted that the items and the scoring procedure of the RFQ-8 can bias the measurement of reflective functioning [11,22]. Namely, items of the RFQ-8 might capture the construct of reflective functioning difficulties insufficiently. For example, hypermentalization (certainty) is only measured by a tendency to disagree with uncertainty, externally oriented and non-behavior-related mentalization capacities might be underrepresented by the items, and multiple items refer on negative urgency-related features. Moreover, as responses on four items are simultaneously weighted on both subscales, the recoding procedure contributes to have redundant variables and subscale scores which are non-independent in confirmatory factor analysis (CFA) [11,22]. Therefore, it was suggested that the structural validity of the RFQ-8 should be examined based on the items with the original response scale. Using this approach, a unidimensional structure of the RFQ was confirmed. Due to the insufficient measurement of hypermentalization (certainty) in the scale, it might be possible that a unidimensional structure of the RFQ-8 can indicate a continuum between low and high levels of hypomentalization (uncertainty) [11,22].

The present study had two main aims. First, to examine the latent structure of the RFQ-8 based on the originally scored items by testing the model fit of competing measurement models in two separate representative samples of adults and young adults. To the best of the Authors’ knowledge, only two studies examined so far the latent structure of the RFQ-8 based on the items with original response scale [11,22]. Except for one research [22], previous studies predominantly analyzed the latent structure of the RFQ-8 based on non-representative samples which can limit the generalizability of the findings. Moreover, there was no attempt in the literature to examine the reproducibility of any factor structure in separate representative samples. Therefore, it was expected that the present study can contribute to enhance the understanding on the latent structure of the RFQ-8 in the general, non-clinical population. Assessing the dimensionality of the RFQ-8 specifically among young adults is important as individuals in the age group can show an increased risk for mental health problems [23].

Second, to examine linear and non-linear associations between mentalization difficulties and maladaptive psychological characteristics, including rumination, worry (both considered as maladaptive emotion regulation forms) [24,25], impulsivity and sensation seeking (both considered as transdiagnostic personality dimensions for psychiatric disorders) [26] and lack of well-being (as an indirect indicator of depressive symptoms) [27]. According to the original, bipolar continuum conceptualization of mentalization difficulties by Fonagy et al. [1], a non-linear relationship can be hypothesized with maladaptive psychological outcomes (e.g., those with high hyper- and hypomentalization can show an increased risk for maladaptive psychological characteristics) [11]. On the other hand, based on the assumed unidimensional structure of the RFQ-8, it is also possible that a linear relationship describes best the relationship with maladaptive psychological outcomes (e.g., as the level of hypomentalization increases, the levels of maladaptive outcomes also increases) [11]. Therefore, by examining simultaneously both linear and quadratic (non-linear) effects of maladaptive psychological characteristics on mentalization difficulties, it is possible to gain further knowledge on how mentalization difficulties are linked to maladaptive psychological outcomes.

## 2. Materials and methods

### 2.1. Participants and procedures

The present cross-sectional study used data from two separate representative samples. On the one hand, the sample from the first data collection round (between March and July 2019) of the Budapest Longitudinal Study (BLS) (https://www.bls2018.hu/main/en) was considered. The target population in the BLS was the young adult (aged between 18–34 years) population from Budapest, the capital city of Hungary. Stratified and random sampling procedure contributed to a representative sample in terms of age and district of residence (net sample size: *N* = 3890; proportion of females: 51.68% [*N* = 2010]; mean age: 27.06 years [*SD* = 4.76]). On the other hand, data (collected between March and April 2019) from the National Survey on Addiction Problems in Hungary 2019 (NSAPH 2019) were also considered in the present study [28]. The NSAPH 2019 used a nationally representative sample of the Hungarian adult population aged between 18–64 years. Stratified and random sampling procedure assured sample representativeness in terms of age, geographic regions and size of residence (net sample size: *N* = 1385; proportion of females: 53.20% [*N* = 737]; mean age: 41.77 years [*SD* = 13.08]). Questionnaires in both studies focused on addictive behaviors and their psychosocial correlates. Individuals provided informed written consent before starting the participation in both studies. Study procedures followed the ethical standards of the Declarations of Helsinki. The study protocols have been approved by the Research Ethics Committee of the Faculty of Education and Psychology of ELTE Eötvös Loránd University (protocol number: 2019/212) and by the Research Ethics Committee of the Medical Research Council (reference number: 60471-2/2018/EKU). The studies were not preregistered.

### 2.2 Measurements

#### 2.2.1. Reflective Functioning Questionnaire (RFQ)

The shortened, 8-item version of the RFQ (RFQ-8) was used to measure difficulties in mentalization [1]. Individuals provided responses for each item on a seven-point scale (1 = Do not agree at all, 7 = Agree completely). Further characteristics of the scale are described in the Introduction. Previous studies have not yet tested the psychometric properties of the Hungarian version of the RFQ-8, this was first done in the present study.

#### 2.2.2. Barratt Impulsiveness Scale (BIS)

The level of trait impulsivity (e.g., cognitive and behavioral impulsivity, impatience/restlessness) was measured by a shortened, 10-item version of the revised, 21-item BIS (BIS-R-21) [29,30]. Respondents evaluated the items of the questionnaire on a four-point scale (1 = Rarely never/Never, 4 = Almost always/Always). A three-factor structure (including factors of cognitive and behavioral impulsivity, impatience/restlessness) was confirmed in Hungarian samples during the development of the BIS-R-21, with acceptable levels of internal reliability, and the validity of the impulsivity factors was also supported via their associations with externalizing characteristics and psychological distress [29]. Total scale score was calculated to represent the overall level of impulsivity. Adequate levels of internal consistency were presented in both samples (Young adults: *α* = 0.78; Adults: *α* = 0.82).

#### 2.2.3. Brief Sensation Seeking Scale (BSSS)

The 8-item BSSS was used to measure the respondents’ sensation seeking tendencies (e.g., thrill-, adventure- and experience seeking, boredom susceptibility, disinhibition) [31,32]. Each item was assessed on a five-point scale (1 = Strongly disagree, 5 = Strongly agree), which allowed to calculate the total score on the scale. In the Hungarian adaptation of the BSSS, the single-factor structure was characterized by adequate fit and internal consistency, while the validity of the scale was supported by its relationships with smoking and smoking expectancies [32]. High rates of internal consistency were shown in both samples (Young adults: *α* = 0.83; Adults: *α* = 0.83).

#### 2.2.4. Ruminative Response Scale (RRS)

The 10-item RRS was used to explore the level of ruminative tendencies (e.g., brooding, reflection) in response to negative affectivity [33,34]. Responses were provided for all items on a four-point scale (1 = Never, 4 = Always). Previous research using the Hungarian version of the 10-item RRS has confirmed the scale’s two-factor structure (including brooding and reflection factors) with adequate internal consistency levels, and the validity of the brooding and reflection subscales has also supported based on their associations with psychological distress [33,35]. Due to the high correlations between the brooding and reflection subscales (*r*≥0.76), the overall level of ruminative thinking was considered based on the total scale score. Very high levels of internal consistency were demonstrated in both samples (Young adults: *α* = 0.91; Adults: *α* = 0.92).

#### 2.2.5. Penn State Worry Questionnaire (PSWQ)

The 3-item, ultra-brief version of the PSWQ was used to measure the tendency for excessive worrying as an emotion regulation strategy [36,37]. A total scale score was calculated based on the 3 items which were assessed on a five-point scale (1 = Not at all typical, 5 = Very typical). The Hungarian validation of the original 16-item PSWQ confirmed a structure with a general worry and two methodological factors, and the internal consistency and validity of the scale (e.g., through its associations with depression and anxiety) were also found to be adequate [37]. Optimal levels of internal consistency were shown in both samples (Young adults: *α* = 0.93; Adults: *α* = 0.91).

#### 2.2.6. World Health Organization Well-Being Index (WHO WBI)

The participants’ general well-being level (as a possible indicator of the absence of depressive symptoms) was evaluated in the past one moths based on the 5-item WHO WBI (WHO WBI-5) [27,38,39]. The respondents evaluated the items of the scale on a four-point scale (0 = Was not typical, 3 = Was very typical) [39]. In the Hungarian adaptation of the WHO WBI-5, the unidimensional structure of the scale was supported with good internal consistency, and the validity of the scale was also found to be acceptable based on the correlations observed with depression, anxiety, hopelessness, life meaningfulness, and subjective health [39]. The total scale score was considered in the analyses. Decreased total scores on the WHO WBI-5 can indicate a risk for major depression [27]. High rates of internal consistency were presented in both samples (Young adults: *α* = 0.88; Adults: *α* = 0.89).

### 2.3. Data analysis

CFA was performed separately among young adult and adults to test the model fit of competing factor models of the RFQ-8. First, the two-factor model of the RFQ-8 was examined. The certainty and uncertainty factors were defined by 6–6 recoded variables with each using a four-point scale (between 0 and 3). These variables were considered as ordered categorical variables, thus the weighted least squares means and variance adjusted (WLSMV) estimation method was applied. However, as scores on some of the indicator items were dependent on each other (i.e., the polychoric correlations between these variables are -1) [11], measurement models of the RFQ-8 based on the items using the original, seven-point response scale were considered primarily.

Two competing measurement models were estimated and compared in terms of model fit, partially building on Müller et al.’s [11] approach: (i) a one-factor model (i.e., the 8 items of the RFQ-8 loaded on a general mentalization difficulties factor), and (ii) a two-factor model (i.e., following the original approach of Fonagy et al. [1], 6–6 items loaded on the correlating certainty and uncertainty factors, thus some of the variables loaded simultaneously on both factors). Each indicator variable in these models were considered as ordered categorical variables, therefore the WLSMV estimation method was applied.

Multiple model fit indices were considered to assess the level of model fit of each model. In the cases of the Comparative Fit Index (CFI) and the Tucker Lewis Index (TLI), values ≥0.900 indicate adequate model fit and values ≥0.950 show optimal model fit. For the Root Mean Square Error of Approximation (RMSEA) adequate model fit is shown by values ≤0.080 and optimal model fit is indicated by values ≤0.050. However, in accordance with literature recommendations, the models were not simply evaluated based on fit indices [40]. The fit indices can indicate a model’s poor fit or inappropriate specification, but they are not suitable on their own to draw conclusions about the acceptability and applicability of a model. Therefore, the most optimal solution was selected not solely on the basis of the model fit indices, but also by interpreting the parameter estimates of a model [40]. Regarding the latter criterion, the models were evaluated in terms of the direction, magnitude and significance of the factor loadings and correlations, taking into account both statistical and theoretical aspects [40]. For statistical aspects, an important evaluation criterion was that the associations between the observed indicators and the latent factors should be meaningful. That is, it was expected that the factor loadings should be significant and at least moderately strong (i.e., *λ*≥0.30). In addition, for CFA parameters, it was also taken into account that they should not show out-of-range values, which could indicate model misspecification or even a nonpositive definite matrix (e.g., with Haywood cases, correlations or loadings above 1.00) [40]. For the theoretical evaluation of parameter estimates, the direction of factor loadings and correlations was evaluated to ensure that they were in line with the a priori assumptions [40]. Finally, for the two-factor model, an important evaluation criterion was whether the discriminant validity of the two latent factors could be supported (e.g., factor correlations above 0.80 may indicate inadequate discriminant validity, with excessive overlap between factors, raising issues about their practical and statistical distinguishability) [40]. McDonald’s omega reliability index (*ω*) was calculated as a measure of internal consistency for the best fitting model.

Next, a hierarchical, multiple indicators multiple causes (MIMIC) model was performed separately among young adults and adults to investigate the associations between the latent factor(s) of mentalization difficulties and maladaptive psychological characteristics. The outcome variable of the MIMIC model was the latent factor(s) of mentalization difficulties based on the best-fitting measurement model. The predictor variables were defined as observed variables and entered in two separate steps. First, the linear predictive effects of impulsivity, sensation seeking, rumination, worry and well-being were examined, while controlling for gender, age, level of education, economically active vs. inactive status (Model 1). Second, in addition to the abovementioned linear predictive effects, the quadratic terms related to impulsivity, sensation seeking, rumination, worry and well-being were also added to the model in order to investigate their non-linear (quadratic) associations with mentalization difficulties (Model 2). The predictor variables of impulsivity, sensation seeking, rumination, worry and well-being were standardized to avoid issues related to high multicollinearity (e.g., suppressor effects). The change in explained variance was also considered between the two steps of the MIMIC model, which could inform about the magnitude of the quadratic (non-linear) effects. The indicator variables of the latent factor(s) of mentalization difficulties were defined as ordered categorical variables in the MIMIC model, therefore the WLSMV estimation method was applied. Model fit of the MIMIC model was assessed based on the abovementioned criteria related to the CFI, the TLI and the RMSEA.

All analyses were performed by using Mplus 8.0 [41] statistical software. Sampling weights were considered during the analyses to ensure representativeness of the samples. Data and codes for analyses for this study are available at https://osf.io/7w2m3/.

## 3. Results

### 3.1. Confirmatory factor analysis (CFA)

Model fit of the different measurement models of the RFQ-8 is summarized in Table 1. The original, two-factor model with recoded items showed optimal model fit among young adults and adequate model fit among adults based on the CFI and the TLI, whereas the RMSEA indicated insufficient model fit in both samples. Extremely high, negative correlations were shown between certainty and uncertainty factors (for factor loadings and correlations in this model, see: S1 Table).

**Table 1:** Model fit of the measurement models of the Reflective Functioning Questionnaire.

Sample	Items (Scale version)	Model	*χ*^*2*^ (df)	CFI	TLI	RMSEA [90% *CI*]
Young adults	12 recoded items (RFQ-8)	Two-factor model: Uncertainty and Certainty factors	2447.734 (53)***	0.966	0.957	0.109 [0.106–0.113]
Adults	12 recoded items (RFQ-8)	Two-factor model: Uncertainty and Certainty factors	977.906 (53)***	0.949	0.936	0.116 [0.109–0.122]
Young adults	8 items with the original response scale (RFQ-8)	One-factor model	864.964 (20)***	0.985	0.979	0.106 [0.100–0.112]
Adults	8 items with the original response scale (RFQ-8)	One-factor model	608.666 (20)***	0.962	0.946	0.150 [0.140–0.160]
Young adults	8 items with the original response scale (RFQ-8)	Two-factor model: Uncertainty and Certainty factors	450.411 (15)***	0.992	0.985	0.088 [0.081–0.095]
Adults	8 items with the original response scale (RFQ-8)	Two-factor model: Uncertainty and Certainty factors	327.340 (15)***	0.980	0.962	0.126 [0.115–0.138]
Young adults	7 items with the original response scale, without Item 7 (RFQ-R-7)	One-factor model	903.139 (14)***	0.983	0.974	0.130 [0.122–0.137]
Adults	7 items with the original response scale, without Item 7 (RFQ-R-7)	One-factor model	383.474 (14)***	0.975	0.963	0.142 [0.130–0.155]
Young adults	7 items with the original response scale, without Item 7 (RFQ-R-7)	Two-factor model: Uncertainty and Certainty factors	414.473 (9)***	0.992	0.982	0.109 [0.100–0.118]
Adults	7 items with the original response scale, without Item 7 (RFQ-R-7)	Two-factor model: Uncertainty and Certainty factors	92.993 (9)***	0.994	0.987	0.085 [0.069–0.100]

Note. *χ*^*2*^ (df): Chi-square test (degrees of freedom). Level of significance

***p<0.001. CFI: Comparative Fit Index. TLI: Tucker Lewis Index. RMSEA [90% *CI*]: Root Mean Square Error of Approximation [90% Confidence Interval]. RFQ-8: Brief, 8-item Reflective Functioning Questionnaire. RFQ-R-7: Revised, 7-item Reflective Functioning Questionnaire.

The one-factor model based on the 8 items of the RFQ-8 with the original response scale presented adequate and optimal rates of model fit in both samples based on the CFI and the TLI, whereas the RMSEA indicated insufficient levels of model fit among both young adults and adults (for factor loadings in this model, see: Table 2). Except for the reversely coded Item 7 (‘I always know what I feel’), items of the RFQ-8 showed moderate-high, positive and significant factor loadings on the mentalization difficulties factor in both samples. Item 7 had significant, but marginal-small factor loadings on the mentalization difficulties factor in both samples (*λ* = 0.08–0.22). The general mentalization factor in the one-factor model of the RFQ-8 presented high internal consistency among both young adults and adults.

**Table 2:** Factor loadings and internal reliability estimates of the one-factor model of the RFQ-8 and the RFQ-R-7.

	Model with 8 items (RFQ-8)		Model without Item 7 (RFQ-R-7)	
	Young adults	Adults	Young adults	Adults
People’s thoughts are a mystery to me	0.513	0.343	0.514	0.353
I don’t always know why I do what I do	0.832	0.753	0.832	0.749
When I get angry I say things without really knowing why I am saying them	0.884	0.867	0.884	0.866
When I get angry I say things that I later regret	0.827	0.794	0.828	0.797
If I feel insecure I can behave in ways that put others’ backs up	0.865	0.860	0.865	0.860
Sometimes I do things without really knowing why	0.854	0.888	0.853	0.886
I always know what I feel (reversed)	0.080	0.216	**-**	-
Strong feelings often cloud my thinking	0.766	0.757	0.767	0.760
Internal reliability estimates: Omega (*ω*)	0.900	0.888	0.924	0.908

Note. Sample size: Young adults–*N* = 3784; adults–*N* = 1307. Values related to the items are standardized factor loadings (*λ*). All factor loadings are significant at least *p*≤0.001 level. RFQ-8: Brief, 8-item Reflective Functioning Questionnaire. RFQ-R-7: Revised, 7-item Reflective Functioning Questionnaire.

By using the 8 items of the RFQ-8 with the original response scale, the two-factor model showed optimal levels of model fit based on the CFI and the TLI, and the RMSEA showed insufficient levels of model fit for the model among young adults as well as among adults (for factor loadings and correlation in this model, see: S2 Table). However, extremely high, positive factor correlations were presented in both samples (*r*≥0.90). This excessive overlap between the two factors raised questions regarding their distinguishability and indicated their insufficient discriminant validity [40]. Regarding factor loadings, both statistical and theoretical problems were identified. Possibly biased, out-of-range factor loadings (*λ*>1.00) were presented in both samples (i.e., Items 4 and 6 among young adults and Item 6 among adults). In line with this, the latent variable covariance matrix was not positive definite for this model among adults due to a negative item residual variance. This may indicate the presence of Haywood cases and biased results, so the interpretation of these findings should be made with great caution and restrictions [40]. In addition, the interpretation of the two latent factors was theoretically difficult. Contrary to prior expectations, Items 4 (’When I get angry, I say things that I later regret’) and 6 (’Sometimes I do things without really knowing why’) presented negative factor loadings on the factors of Uncertainty and Certainty, respectively. This makes the content of the two factors ambiguous, as items that are related and overlapping in content presented both positive and negative loadings on the same factor on both samples (e.g., Item 6 presented a negative loading on the factor Certainty, while Item 2 [I don’t always know why I do what I do] positively loaded on this factor). As with the one-factor model, Item 7 had significant but marginal-small factor loadings on the Uncertainty factor in both samples (*λ* = 0.08–0.22). Overall, although the fit indices were found to be better for the two-factor model (compared to the one-factor model), statistical and theoretical problems emerged in the evaluation of the parameter estimates, which raised questions regarding the structural and discriminant validity of the two-factor solution. Thus, this model was not considered further.

Due to the low factor loadings of Item 7 in all of the tested models with the items using the original response scale, modified measurement models were constructed by excluding Item 7. This version of the scale was named as Reflective Functioning Questionnaire–Revised– 7 (RFQ-R-7) (see: S1 Appendix). Model fit of these models with 7 items using the original response scale is shown in Table 1. The one-factor model had optimal levels of model fit based on the CFI and the TLI, and insufficient model fit as per the RMSEA in both samples (for factor loadings in this model, see: Table 2). All factor loadings were positive, strong and significant among young adults. In the adult sample, Item 1 (‘People’s thoughts are a mystery to me’) had a positive, moderately strong and significant loading on the latent factor, whereas the other items showed positive, strong and significant relationships with the general mentalization difficulties factor. The general mentalization factor in the one-factor model of the RFQ-R-7 presented high internal consistency in both samples.

In both samples, the two-factor model presented optimal levels of model fit based on the CFI and the TLI, and insufficient levels of model fit based on the RMSEA. However, similar statistical issues were presented for the two-factor model of the RFQ-R-7 using the original response scale than its 8-item alternative (S2 Table). Extremely high positive factor correlations were presented in both samples (*r*≥0.92). This excessive overlap between the two factors indicated insufficient discriminant validity for the Certainty and Uncertainty factors. Therefore, this may suggest that it may be more appropriate to combine the two factors and work with the more parsimonious single-factor solution [40]. Both statistical and theoretical problems were highlighted in the evaluation of the factor loadings. Possibly biased, out-of-range factor loadings (*λ*>1.00) were presented in both samples (i.e., Items 4 and 6 among young adults and Item 6 among adults). In line with this, the latent variable covariance matrix was not positive definite for this model among adults due to a negative item residual variance. This may indicate the presence of Haywood cases and biased results, so the interpretation of these findings should be made with great caution and restrictions [40]. Moreover, Items 2 and 4 showed non-significant factor loadings on the factors of Certainty and Uncertainty, respectively. In addition, the interpretation of the two latent factors was theoretically difficult. Contrary to prior expectations, Items 4 and 6 presented negative factor loadings on the factors of Uncertainty and Certainty, respectively. This makes the content of the two factors ambiguous, as items that are related and overlapping in content presented both positive and negative loadings on the same factor on both samples (e.g., Item 6 presented a negative loading on the factor Certainty, while Item 2 positively loaded on this factor). Overall, in the case of the RFQ-R-7, although the fit indices were found to be better for the two-factor model (compared to the one-factor model), statistical and theoretical problems emerged in the evaluation of the factor loadings and correlations, which raised questions regarding the structural and discriminant validity of the two-factor solution. Thus, this model was also not considered further.

Overall, the one-factor model of the RFQ-R-7 with a general mentalization difficulties factor was considered as the most optimal measurement model in both samples. The CFI and the TLI indicated high levels of model fit for this model in both samples, with moderate-high factor loadings and high internal consistencies. Although the RMSEA showed inadequate model fit among both young adults and adults, it is important to note that for models with low degrees of freedom (df, models of the RFQ-R-7 with originally scored items: df = 9–14) the RMSEA can show insufficient level of model fit–even with very high average factor loadings [42,43]. Therefore, it might be possible that the other fit indices (i.e., CFI, TLI) provided more accurate estimation of the model fit in the current analyses. Although the two-factor model of the RFQ-R-7 was characterized by closer fit to the data than the one-factor model of the RFQ-R-7, the interpretation of the parameter estimates in the two-factor model raised concerns regarding its acceptability and applicability. Among the tested models with the original response scale, the parameter estimates only showed sufficient characteristics in the one-factor model of the RFQ-R-7. For the other models, non-significant and/or low factor loadings were found (i.e., the one-factor model of the RFQ-8 in both samples, and the two-factor model of the RFQ-8 and the RFQ-R-7 in both samples), as well as Haywood cases (i.e., out-of-range factor loadings with *λ*>1.00) and a content ambiguous pattern of factor loadings (i.e., content similar items simultaneously showed positive and negative loadings on the same factor) were presented for the two-factor model of the RFQ-8 and the RFQ-R-7. The extremely low discriminant validity of the Certainty and Uncertainty factors in the two-factor model of the RFQ-8 and the RFQ-R-7 also suggested that it might be worth retaining a more parsimonious singe-factor solution [40]. Therefore, the one-factor model of the RFQ-R-7 was retained for the MIMIC model.

### 3.2. Multiple indicators multiple causes (MIMIC) model

Predictive effects in the MIMIC model in both samples are summarized in Table 3. In the young adult sample, Model 1 (*χ*^*2*^ [68] = 925.240; *p*<0.001; CFI = 0.962; TLI = 0.953; RMSEA [90% *CI*] = 0.060 [0.056–0.063]) as well as Model 2 (*χ*^*2*^ [98] = 1058.441; *p*<0.001; CFI = 0.955; TLI = 0.946; RMSEA [90% *CI*] = 0.053 [0.050–0.056]) showed adequate and optimal levels of model fit. In the first step, higher levels of mentalization difficulties were significantly and positively associated with economically active status, impulsivity, sensation seeking, rumination, worry and well-being. However, the significant effects of economically active vs. inactive status and well-being had marginal effect sizes (*β*<0.10). Impulsivity showed strong association with the general mentalization difficulties factor, while the other significant relationships were weak. In the second step, the abovementioned significant (linear) predictive effects remained significant and had similar effect sizes than in Model 1. In addition to these, the quadratic terms related to impulsivity, sensation seeking, rumination, and worry were also identified as significant. These non-linear associations with general mentalization difficulties are shown in S1–S4 Figs. Overall, the quadratic relationships closely followed the linear associations between the variables. In line with this, only marginal increase (1%) was shown in the overall level of explained variance of the outcome between Models 1 and 2. The significant quadratic effects of impulsivity (S1 Fig), sensation seeking (S2 Fig) and worry (S4 Fig) indicated a possible plateau effect (i.e., after reaching certain values of the predictor variables, less marked increases in mentalization difficulties were observed), whereas the significant quadratic effect of rumination (S3 Fig) highlighted a possible threshold effect (i.e., after reaching certain values of the predictor variable, more sharped increases in mentalization difficulties were observed).

**Table 3:** Predictive effects on the latent factor of mentalization difficulties assessed by the RFQ-R-7.

	Young adults		Adults	
	Model 1 *β* (*S*.*E*.)	Model 2 *β* (*S*.*E*.)	Model 1 *β* (*S*.*E*.)	Model 2 *β* (*S*.*E*.)
Female gender (vs. male gender)	0.024 (0.014)	0.021 (0.013)	-0.002 (0.024)	-0.003 (0.024)
Age	-0.017 (0.014)	-0.019 (0.014)	0.018 (0.026)	0.021 (0.026)
Level of education (years of studying)	-0.003 (0.015)	0.002 (0.015)	0.033 (0.026)	0.029 (0.026)
Economically active status (vs. economically inactive status)	0.084 (0.015)***	0.078 (0.015)***	0.003 (0.023)	-0.012 (0.024)
Impulsivity	0.511 (0.015)***	0.505 (0.014)***	0.422 (0.024)***	0.425 (0.025)***
Sensation seeking	0.125 (0.014)***	0.146 (0.015)***	0.099 (0.026)***	0.135 (0.032)***
Rumination	0.173 (0.015)***	0.137 (0.020)***	0.258 (0.027)***	0.231 (0.033)***
Worry	0.195 (0.015)***	0.272 (0.023)***	0.215 (0.027)***	0.262 (0.035)***
Well-being	0.033 (0.014)*	0.031 (0.015)*	-0.032 (0.026)	-0.062 (0.027)*
Quadratic term: impulsivity	**-**	-0.053 (0.014)***	**-**	-0.037 (0.024)
Quadratic term: sensation seeking	**-**	-0.063 (0.014)***	**-**	-0.052 (0.026)*
Quadratic term: rumination	**-**	0.039 (0.019)*	**-**	0.046 (0.030)
Quadratic term: worry	**-**	-0.092 (0.023)***	**-**	-0.069 (0.033)*
Quadratic term: well-being	**-**	0.027 (0.016)	**-**	-0.047 (0.024)
Explained variance (R^2^)	53%	54%	56%	58%

Notes. Sample size: Young adults–*N* = 3528; adults–*N* = 1171. *β* (*S*.*E*.): Standardized regression coefficient (standard error). Level of significance

*p<0.050

**p<0.010

***p<0.001.

Model 1 (*χ*^*2*^ [68] = 406.152; *p*<0.001; CFI = 0.930; TLI = 0.914; RMSEA [90% *CI*] = 0.065 [0.059–0.071]) and Model 2 (*χ*^*2*^ [98] = 440.597; *p*<0.001; CFI = 0.927; TLI = 0.911; RMSEA [90% *CI*] = 0.055 [0.050–0.060]) demonstrated adequate levels of model fit among adults. In the first step, elevated levels of impulsivity (with moderate effect size), sensation seeking, rumination and worry (with weak effect sizes) significantly and positively predicted general mentalization difficulties. These significant (linear) relationships remained significant in the second step as well with similar effect sizes compared to Model 1. Moreover, there was a significant and negative (linear) relationship between well-being and mentalization difficulties, though with marginal effect size. The quadratic terms related to sensation seeking and worry were also significant. These non-linear associations with general mentalization difficulties are shown in S5 and S6 Figs. Overall, there were only minor differences between the linear and quadratic trend lines in these associations. In line with this, only marginal increase (2%) was shown in the overall level of explained variance of the outcome between Models 1 and 2. The significant quadratic effects of sensation seeking (S5 Fig) indicated a possible threshold effect, whereas the significant quadratic effect of worry (S6 Fig) highlighted a possible plateau effect.

## 4. Discussion

The present study aimed to examine further the latent dimensionality of the RFQ-8 in two separate representative samples of young adults and adults. CFA was used to test the model fit of competing measurement models of the RFQ-8. Considering the limitations of the recoding procedure regarding the calculation of the subscales of the RFQ-8, the analyses primarily examined measurement models with items of the RFQ-8 using the original, seven-point response scale. The present analyses revealed that Item 7 (‘I always know what I feel’) shows only weak associations with any factor of mentalization difficulties. Therefore, it was suggested that more precise measurement of mentalization difficulties can be obtained by using a revised, seven-item version of the brief RFQ, without Item 7 (named as the Reflective Functioning Questionnaire–Revised– 7, RFQ-R-7) (see: S1 Appendix). A previous study using representative adult sample from Germany also reported insufficient psychometric properties for Item 7 [22]. This pattern might be explained by the difference in formulation for Item 7 compared to the other items of the RFQ-8. Higher agreement with this item refers to a certainty of understanding internal mental states, whereas stronger agreement with the other items of RFQ-8 can indicate tendencies of uncertainty of understanding internal mental states.

Overall, a one-factor model with a general mentalization difficulties factor showed the most optimal levels of model fit in both samples. In the case of the RFQ-R-7, each item had significant, moderate-strong and positive associations with the general mentalization difficulties factor. Moreover, the general factor was also characterized by high levels of internal consistency in both samples. These findings suggested that a unidimensional latent structure can describe most optimally the covariance between the items of the RFQ-R-7. Previous studies which performed CFA by using the items of the RFQ-8 with the original response scale also confirmed unidimensional structures for the scale [11,22]. Overall, the present findings might add to the current understanding on the latent structure of the scale by examining that in a new cultural context (i.e., among Hungarian individuals, previous studies with similar analytical approach included participants from Germany and the United States) and by confirming the replicability of the one-factor structure in two separate representative samples of adults and young adults.

However, it is important to note that for both the RFQ-8 and the RFQ-R-7, the two-factor model showed a better fit to the data than the single-factor model. However, the statistical (e.g., Haywood cases with out-of-range factor loadings, non-distinguishable factors due to the extremely high correlations between them) and theoretical problems (e.g., not straightforward factor content due to positive and negative loadings on the same factor by content similar items) associated with the parameter estimates in the two-factor models did not allow for the acceptance and applicability of these models. Future research would be needed to test the validity of these models on other samples or possibly suggest revisions to these models.

Next, linear and non-linear associations were examined between the mentalization difficulties factor of the RFQ-R-7 and maladaptive psychological characteristics. Impulsivity, sensation seeking, rumination and worry consistently presented significant, positive and at least weak linear associations with general mentalization difficulties in both samples of young adults and adults. Among these maladaptive psychological characteristics, impulsivity showed the strongest association with mentalizing difficulties, with strong and moderately strong associations among young adults and adults, respectively.

Both impulsivity and sensation seeking are externalizing personality traits which are considered as transdiagnostic risk factors for externalizing psychopathologies [26,44–46]. Previous studies also reported positive correlations between impulsivity and mentalization difficulties [10,11,15], whereas limited data are available on the link between sensation seeking and difficulties in reflective functioning. The co-occurrence of high levels of impulsivity, sensation seeking and mentalization difficulties might be explained by the preference for disinhibition and lack of premeditation across these psychological constructs (e.g., the item of “Sometimes I do things without really knowing why” and “I don’t always know why I do what I do” from the RFQ-R-7 show overlap with impulsivity and sensation seeking). Impulsive behaviors may occur when a person does not adequately consider and plan how their behavior may have behavioral and mental consequences (e.g., emotions, thoughts) for themselves and others (i.e., hypomentalization). In addition, in the presence of mentalizing impairments, impulsive and unrealistic assumptions about the thoughts and feelings of others may often be raised (i.e. hypermentalization) [17]. Moreover, negative urgency-related characteristics are considered as forms of impulsivity as well as reflective functioning difficulties according to the RFQ-R-7 [11]. Individuals with high sensation seeking can also present impairments in terms of emotion regulation, specifically problems in regulating positive emotions [16]. That is, general emotion regulation difficulties also may account for the positive link between mentalization difficulties and externalizing personality traits of impulsivity and sensation seeking.

Both rumination and worry can be conceptualized as maladaptive emotion regulation forms as they can show positive relationships with adverse outcomes of mental health [24,25]. Therefore, the co-occurrence of high levels of rumination, worry and mentalization difficulties is in accordance with those previous findings which showed positive correlations between difficulties in emotion regulation, maladaptive emotion regulation strategies and impairments in reflective functioning [2,10–14]. The positive correlations between these variables might be accounted for the overlap between the items of the RFQ-R-7 and general emotion regulation difficulties (e.g., the item of “People’s thoughts are a mystery to me”), whereas other items of the RFQ-R-7 show similar characteristics to rumination and worry (e.g., the item of “Strong feelings often cloud my thinking”). Furthermore, a possible explanation for the positive associations between mentalizing difficulties and repetitive negative thinking styles (i.e., rumination and worry) may be that affected individuals may have a general difficulty thinking about mental states. For example, previous research has indicated that metacognitive beliefs may be associated with the use of maladaptive emotion regulation strategies such as rumination and worry [47,48]. Metacognitive beliefs (e.g., positive beliefs about the benefits of using worry and rumination, negative beliefs about the uncontrollability and dangers of thoughts) overlap with the concept of mentalization: they also allow understanding of others’ and one’s own mental states and their motivations, and foster taking the perspective of others [49].

The validation analyses of the RFQ-R-7 also revealed significant quadratic (non-linear) effects, for example, with sensation seeking and worry in both samples. However, the significant quadratic relationships closely followed the linear associations between the variables (e.g., suggesting marginal plateau and threshold effects). In line with this, only marginal increases were shown in the level of explained variance of mentalization difficulties due to the inclusion of the quadratic effects compared with the model containing only the linear predictive effects. In line with previous literature, these findings may suggest that the general mentalization difficulties factor in the unidimensional structure of the RFQ-8 and the RFQ-R-7 can indicate a continuum between low and high levels of hypomentalization (uncertainty), while hypermentalization (certainty) is measured insufficiently in these scales [11,22]. That is, based on the assumed unidimensional structure of the RFQ-R-7, it may be possible that an approximately linear relationship describes best the relationship with maladaptive psychological outcomes (e.g., as the level of hypomentalization increases, the levels of maladaptive outcomes also increases) [11]. In other words, increasing scores on items of the RFQ-R-7 rated on the original seven-point scale may indicate increasing hypomentalization, meaning that low scores may represent more adaptive psychological functioning, while higher scores may imply more maladaptive psychological functioning. On the other hand, the general mentalizing difficulties (hypomentalization) factor measured by the RFQ-R-7 version is probably not suitable to test the Fonagy et al.’s concept of the bipolar continuum of mentalizing difficulties [1]. Namely, a non-linear relationship can be hypothesized with maladaptive psychological outcomes: on some of the items low scores can represent hypermentalization (certainty), whereas high scores can indicate hypomentalization (uncertainty); and those with high hyper- and hypomentalization can show an increased risk for maladaptive psychological characteristics [1,11].

### 4.1. Limitations

Cautious interpretation of the present findings is warranted due to methodological limitations. First, the cross-sectional design of the study did not allow to test longitudinal invariance of the confirmed unidimensional structure of the questionnaire as well as causal relationships between mentalization difficulties and the validating psychological variables. Second, due to the applied self-report measurement it might be possible that differences in mentalization difficulties were not captured accurately (e.g., automatic and fluctuating mentalization processes), whereas it is also possible that social desirability effects influenced the participants’ responses. Third, the RFQ-8 and the RFQ-R-7 only provided a superficial assessment for reflective functioning (e.g., measuring mostly hypomentalization tendencies regarding the individual’s own behavior), thus it was not possible to explore the multidimensional nature of mentalization (i.e., other mentalization questionnaires were not included in the study questionnaires). That is, the present study only provided data specifically for the dimensionality of the RFQ-8 and not for mentalization difficulties in general. Moreover, the present study missed to analyze the association between the RFQ-R-7 and other, mentalization-based measures. Fourth, it is also possible that relevant psychological predictors were not included in the multivariate MIMIC analyses which might influenced the findings (e.g., psychopathological symptom levels of borderline personality disorder or major depressive disorder, other emotion regulation strategies and personality traits). Finally, total scale scores were calculated for each psychological predictor of mentalization difficulties. It might be possible that by using subscale scores (e.g., cognitive and behavioral impulsivity, impatience/restlessness in the case of the BIS-R-21), more detailed associations would have been revealed between difficulties in reflective functioning and the other psychological constructs.

## 5. Conclusions

In line with previous literature data, the present study suggested that it is preferable to use the original response scale of the RFQ-8 to measure the level of difficulties in reflective functioning [11,22]. Moreover, CFA underlined that more precise measurement of mentalization difficulties can be obtained by using, a modified, seven-item version of the brief RFQ, without Item 7 (RFQ-R-7). A unidimensional model with a general mentalization difficulties factor provided the most optimal level of model fit and psychometric characteristics. This model indicated that future studies might consider using the total scale score. However, the interpretation of the low and high scores on the general mentalization difficulties factor (i.e., the total score of the RFQ-R-7) might be equivocal. It might be possible that this dimension predominantly captures difficulties of hypomentalization (uncertainty), thus low and high scores on the RFQ-R-7 represent the absence and presence of hypomentalization difficulties, respectively. Alternatively, in line with the original conceptualization of the scoring of the RFQ-8, low and high scores on the RFQ-R-7 might represent high rates of hypermentalization (certainty) and hypomentalization (uncertainty), respectively.

Overall, the present findings suggested that the RFQ-8 and the RFQ-R-7 provide measurement for hypomentalization difficulties, whereas the construct of hypermentalization is assessed only limitedly with these set of items. Specifically, associations between mentalization difficulties and maladaptive psychological characteristics pointed out that low scores on the RFQ-R-7 are associated with more adaptive psychological characteristics (e.g., lower levels of impulsivity, sensation seeking, rumination, worry). That is, in accordance with previous findings [11,22], these data might indicate that lower scores on some of the items of the RFQ most likely are not signs of a maladaptive, certainty-related reflective functioning (i.e., hypermentalization). In other words, it might possible that the total score on the RFQ-R-7 captures a dimension of hypomentalization ranging between low and high levels of difficulties regarding uncertainty. Previous findings also revealed that the measurement of hypermentalization is only limitedly possible by using the RFQ-8 [11,22]. Therefore, future studies might consider revising the brief version of the RFQ to contain items which are more suitable to measure hypermentalization.

## Supporting information

S1 Fig Linear and non-linear relationship between impulsivity and mentalization difficulties among young adults. Notes: the solid line shows the quadratic correlation between the variables, and the dashed line shows the linear correlation between the variables.
(DOCX)

S2 Fig Linear and non-linear relationship between sensation seeking and mentalization difficulties among young adults. Notes: the solid line shows the quadratic correlation between the variables, and the dashed line shows the linear correlation between the variables.
(DOCX)

S3 Fig Linear and non-linear relationship between rumination and mentalization difficulties among young adults. Notes: the solid line shows the quadratic correlation between the variables, and the dashed line shows the linear correlation between the variables.
(DOCX)

S4 Fig Linear and non-linear relationship between worry and mentalization difficulties among young adults. Notes: the solid line shows the quadratic correlation between the variables, and the dashed line shows the linear correlation between the variables.
(DOCX)

S5 Fig Linear and non-linear relationship between sensation seeking and mentalization difficulties among adults. Notes: the solid line shows the quadratic correlation between the variables, and the dashed line shows the linear correlation between the variables.
(DOCX)

S6 Fig Linear and non-linear relationship between worry and mentalization difficulties among adults. Notes: the solid line shows the quadratic correlation between the variables, and the dashed line shows the linear correlation between the variables.
(DOCX)

S1 Table Factor loadings of the two-factor model by using recoded items. (DOCX)

S2 Table Factor loadings of the two-factor model by using the original response scales. (DOCX)

S1 Appendix The Reflective Functioning Questionnaire–Revised– 7 (RFQ-R-7). (DOCX)

## References

[1] Fonagy P, Luyten P, Moulton-Perkins A, Lee YW, Warren F, Howard S, et al. Development and Validation of a Self-Report Measure of Mentalizing: The Reflective Functioning Questionnaire. Laws K, editor. PLOS ONE. 2016 Jul 8;11(7):e0158678. doi: 10.1371/journal.pone.0158678 27392018 PMC4938585

[2] Luyten P, Campbell C, Allison E, Fonagy P. The Mentalizing Approach to Psychopathology: State of the Art and Future Directions. Annu Rev Clin Psychol. 2020 May 7;16(1):297–325. doi: 10.1146/annurev-clinpsy-071919-015355 32023093

[3] American Psychiatric Association. Diagnostic and statistical manual of mental disorders (5th ed.). Washington, DC: Author; 2013.

[4] Chung YS, Barch D, Strube M. A Meta-Analysis of Mentalizing Impairments in Adults With Schizophrenia and Autism Spectrum Disorder. Schizophr Bull. 2014 May;40(3):602–16. doi: 10.1093/schbul/sbt048 23686020 PMC3984506

[5] Katznelson H. Reflective functioning: A review. Clin Psychol Rev. 2014 Mar;34(2):107–17. doi: 10.1016/j.cpr.2013.12.003 24486522

[6] Simonsen CB, Jakobsen AG, Grøntved S, Kjaersdam Telléus G. The mentalization profile in patients with eating disorders: a systematic review and meta-analysis. Nord J Psychiatry. 2020 Jun 26;74(5):311–22. doi: 10.1080/08039488.2019.1707869 31910059

[7] Rossouw TI, Fonagy P. Mentalization-Based Treatment for Self-Harm in Adolescents: A Randomized Controlled Trial. J Am Acad Child Adolesc Psychiatry. 2012 Dec;51(12):1304–1313.e3. doi: 10.1016/j.jaac.2012.09.018 23200287

[8] Vogt KS, Norman P. Is mentalization‐based therapy effective in treating the symptoms of borderline personality disorder? A systematic review. Psychol Psychother Theory Res Pract. 2019 Dec;92(4):441–64. doi: 10.1111/papt.12194 30099834 PMC6900007

[9] Volkert J, Hauschild S, Taubner S. Mentalization-Based Treatment for Personality Disorders: Efficacy, Effectiveness, and New Developments. Curr Psychiatry Rep. 2019 Apr;21(4):25. doi: 10.1007/s11920-019-1012-5 30852694

[10] Euler S, Nolte T, Constantinou M, Griem J, Montague PR, Fonagy P, et al. Interpersonal Problems in Borderline Personality Disorder: Associations With Mentalizing, Emotion Regulation, and Impulsiveness. J Personal Disord. 2021 Apr;35(2):177–93. doi: 10.1521/pedi_2019_33_427 30920937

[11] Müller S, Wendt LP, Spitzer C, Masuhr O, Back SN, Zimmermann J. A Critical Evaluation of the Reflective Functioning Questionnaire (RFQ). J Pers Assess. 2021 Oct 1;1–15. 10.1080/00223891.2021.1981346 34597256

[12] Schwarzer NH, Nolte T, Fonagy P, Gingelmaier S. Mentalizing and emotion regulation: Evidence from a nonclinical sample. Int Forum Psychoanal. 2021 Jan 2;30(1):34–45.

[13] Sharp C, Pane H, Ha C, Venta A, Patel AB, Sturek J, et al. Theory of Mind and Emotion Regulation Difficulties in Adolescents With Borderline Traits. J Am Acad Child Adolesc Psychiatry. 2011 Jun;50(6):563–573.e1. doi: 10.1016/j.jaac.2011.01.017 21621140

[14] Vahidi E, Ghanbari S, Behzadpoor S. The relationship between mentalization and borderline personality features in adolescents: mediating role of emotion regulation. Int J Adolesc Youth. 2021 Jan 1;26(1):284–93.

[15] Cosenza M, Ciccarelli M, Nigro G. The steamy mirror of adolescent gamblers: Mentalization, impulsivity, and time horizon. Addict Behav. 2019 Feb;89:156–62. doi: 10.1016/j.addbeh.2018.10.002 30316141

[16] Rogier G, Velotti P. Conceptualizing gambling disorder with the process model of emotion regulation. J Behav Addict. 2018 Jun;7(2):239–51. doi: 10.1556/2006.7.2018.52 29936851 PMC6174584

[17] Bateman A, Fonagy P. Mentalization-based treatment for personality disorders: a practical guide. First edition. Oxford: Oxford University Press; 2016. 468 p.

[18] Badoud D, Luyten P, Fonseca-Pedrero E, Eliez S, Fonagy P, Debbané M. The French Version of the Reflective Functioning Questionnaire: Validity Data for Adolescents and Adults and Its Association with Non-Suicidal Self-Injury. Tran US, editor. PLOS ONE. 2015 Dec 29;10(12):e0145892. doi: 10.1371/journal.pone.0145892 26714319 PMC4694697

[19] Griva F, Pomini V, Gournellis R, Doumos G, Thomakos P, Vaslamatzis G. Psychometric properties and factor structure of the Greek version of Reflective Functioning Questionnaire. Psychiatriki. 2020 Oct 1;31(3):216–24. doi: 10.22365/jpsych.2020.313.216 33099462

[20] Morandotti N, Brondino N, Merelli A, Boldrini A, De Vidovich GZ, Ricciardo S, et al. The Italian version of the Reflective Functioning Questionnaire: Validity data for adults and its association with severity of borderline personality disorder. Fonseca-Pedrero E, editor. PLOS ONE. 2018 Nov 1;13(11):e0206433. doi: 10.1371/journal.pone.0206433 30383803 PMC6211684

[21] Seyed Mousavi PS, Vahidi E, Ghanbari S, Khoshroo S, Sakkaki SZ. Reflective Functioning Questionnaire (RFQ): Psychometric Properties of the Persian Translation and Exploration of Its Mediating Role in the Relationship between Attachment to Parents and Internalizing and Externalizing Problems in Adolescents. J Infant Child Adolesc Psychother. 2021 Jul 3;20(3):313–30.

[22] Spitzer C, Zimmermann J, Brähler E, Euler S, Wendt L, Müller S. Die deutsche Version des Reflective Functioning Questionnaire (RFQ): Eine teststatistische Überprüfung in der Allgemeinbevölkerung. PPmP—Psychother · Psychosom · Med Psychol. 2021 Mar;71(03/04):124–31. 10.1055/a-1234-6317 33063306

[23] Mojtabai R, Olfson M, Han B. National Trends in the Prevalence and Treatment of Depression in Adolescents and Young Adults. Pediatrics. 2016 Dec 1;138(6):e20161878. doi: 10.1542/peds.2016-1878 27940701 PMC5127071

[24] Aldao A, Nolen-Hoeksema S, Schweizer S. Emotion-regulation strategies across psychopathology: A meta-analytic review. Clin Psychol Rev. 2010 Mar;30(2):217–37. doi: 10.1016/j.cpr.2009.11.004 20015584

[25] Salters-Pedneault K, Roemer L, Tull MT, Rucker L, Mennin DS. Evidence of Broad Deficits in Emotion Regulation Associated with Chronic Worry and Generalized Anxiety Disorder. Cogn Ther Res. 2006 Dec 4;30(4):469–80.

[26] Kotov R, Krueger RF, Watson D, Achenbach TM, Althoff RR, Bagby RM, et al. The Hierarchical Taxonomy of Psychopathology (HiTOP): A dimensional alternative to traditional nosologies. J Abnorm Psychol. 2017;126(4):454–77. doi: 10.1037/abn0000258 28333488

[27] Topp CW, Østergaard SD, Søndergaard S, Bech P. The WHO-5 Well-Being Index: A Systematic Review of the Literature. Psychother Psychosom. 2015;84(3):167–76. doi: 10.1159/000376585 25831962

[28] Paksi B, Pillok P, Magi A, Demetrovics Z, Felvinczi K. [The National Survey on Addiction Problems in Hungary 2019 (NSAPH): Methodology and sample description]. Neuropsychopharmacol Hung. 2021 Mar;23(1):184–207. 33835041

[29] Kapitány-Fövény M, Urbán R, Varga G, Potenza MN, Griffiths MD, Szekely A, et al. The 21-item Barratt Impulsiveness Scale Revised (BIS-R-21): An alternative three-factor model. J Behav Addict. 2020 Jun;9(2):225–46. doi: 10.1556/2006.2020.00030 32609636 PMC8939423

[30] Patton JH, Stanford MS, Barratt ES. Factor structure of the barratt impulsiveness scale. J Clin Psychol. 1995 Nov;51(6):768–74. doi: 10.1002/1097-4679(199511)51:6&lt;768::aid-jclp2270510607&gt;3.0.co;2-1 8778124

[31] Hoyle RH, Stephenson MT, Palmgreen P, Lorch EP, Donohew RL. Reliability and validity of a brief measure of sensation seeking. Personal Individ Differ. 2002 Feb;32(3):401–14.

[32] Urbán R. Smoking outcome expectancies mediate the association between sensation seeking, peer smoking, and smoking among young adolescents. Nicotine Tob Res. 2010 Jan;12(1):59–68. doi: 10.1093/ntr/ntp174 19959571 PMC2802571

[33] Kokonyei G, Szabo E, Kocsel N, Edes A, Eszlari N, Pap D, et al. Rumination in migraine: Mediating effects of brooding and reflection between migraine and psychological distress. Psychol Health. 2016 Dec;31(12):1481–97. doi: 10.1080/08870446.2016.1235166 27616579 PMC5062042

[34] Treynor W, Gonzalez R, Nolen-Hoeksema S. Rumination reconsidered: A psychometric analysis. Cogn Ther Res. 2003;27(3):247–59.

[35] Eszlari N, Kovacs D, Petschner P, Pap D, Gonda X, Elliott R, et al. Distinct effects of folate pathway genes MTHFR and MTHFD1L on ruminative response style: a potential risk mechanism for depression. Transl Psychiatry. 2016 Mar;6(3):e745–e745. doi: 10.1038/tp.2016.19 26926881 PMC4872445

[36] Berle D, Starcevic V, Moses K, Hannan A, Milicevic D, Sammut P. Preliminary validation of an ultra-brief version of the Penn State Worry Questionnaire. Clin Psychol Psychother. 2011 Jul;18(4):339–46. doi: 10.1002/cpp.724 20806422

[37] Pajkossy P, Simor P, Szendi I, Racsmány M. Hungarian Validation of the Penn State Worry Questionnaire (PSWQ): Method Effects and Comparison of Paper-Pencil Versus Online Administration. Eur J Psychol Assess. 2015 Jul;31(3):159–65.

[38] Bech P, Gudex C, Johansen S. The WHO (Ten) Weil-Being Index: Validation in Diabetes. Psychother Psychosom. 1996;65(4):183–90. 8843498 10.1159/000289073

[39] Susánszky É, Konkoly Thege B, Stauder A, Kopp M. A WHO jól-lét kérdőív rövidített (WBI-5) magyar változatának validálása a Hungarostudy 2002 országos lakossági egészségfelmérés alapján. Mentálhig És Pszichoszomatika. 2006 Sep;7(3):247–55.

[40] Brown TA. Confirmatory factor analysis for applied research. Second edition. New York; London: The Guilford Press; 2015. 462 p. (Methodology in the social sciences).

[41] Muthén LK, Muthén BO. Mplus User’s Guide. Eight Edition. Los Angeles, CA: Muthén & Muthén; 2017.

[42] Kenny DA, Kaniskan B, McCoach DB. The Performance of RMSEA in Models With Small Degrees of Freedom. Sociol Methods Res. 2015 Aug;44(3):486–507.

[43] Shi D, Lee T, Maydeu-Olivares A. Understanding the Model Size Effect on SEM Fit Indices. Educ Psychol Meas. 2019 Apr;79(2):310–34. doi: 10.1177/0013164418783530 30911195 PMC6425088

[44] Coskunpinar A, Dir AL, Cyders MA. Multidimensionality in Impulsivity and Alcohol Use: A Meta-Analysis Using the UPPS Model of Impulsivity. Alcohol Clin Exp Res. 2013 Sep;37(9):1441–50. doi: 10.1111/acer.12131 23578176 PMC3732812

[45] Lauriola M, Panno A, Levin IP, Lejuez CW. Individual Differences in Risky Decision Making: A Meta-analysis of Sensation Seeking and Impulsivity with the Balloon Analogue Risk Task: Personality and Risky Decision Making. J Behav Decis Mak. 2014 Jan;27(1):20–36.

[46] VanderVeen JD, Hershberger AR, Cyders MA. UPPS-P model impulsivity and marijuana use behaviors in adolescents: A meta-analysis. Drug Alcohol Depend. 2016 Nov;168:181–90. doi: 10.1016/j.drugalcdep.2016.09.016 27682358

[47] Wells A, Matthews G. Modelling cognition in emotional disorder: The S-REF model. Behav Res Ther. 1996 Nov;34(11–12):881–8. doi: 10.1016/s0005-7967(96)00050-2 8990539

[48] Spada MM, Nikčević AV, Kolubinski DC, Offredi A, Giuri S, Gemelli A, et al. Metacognitions, rumination, and worry in personality disorder. J Affect Disord. 2021 Oct;293:117–23. doi: 10.1016/j.jad.2021.06.024 34175593

[49] Dimaggio G, Lysaker PH. Metacognition and Mentalizing in the Psychotherapy of Patients With Psychosis and Personality Disorders: Metacognition and Mentalizing. J Clin Psychol. 2015 Feb;71(2):117–24. 25557317 10.1002/jclp.22147

