# Supplementary material for: The Reflective Functioning Questionnaire–Revised– 7 (RFQ-R-7): A new measurement model assessing hypomentalization
Source: PLoS One. 2023 Feb 24;18(2):e0282000. doi: 10.1371/journal.pone.0282000 (PMC9956064; doi:10.1371/journal.pone.0282000)
Supplement: S4 Fig — Notes: the solid line shows the quadratic correlation between the variables, and the dashed line shows the linear correlation between the variables. (DOCX) [file pone.0282000.s004.docx]

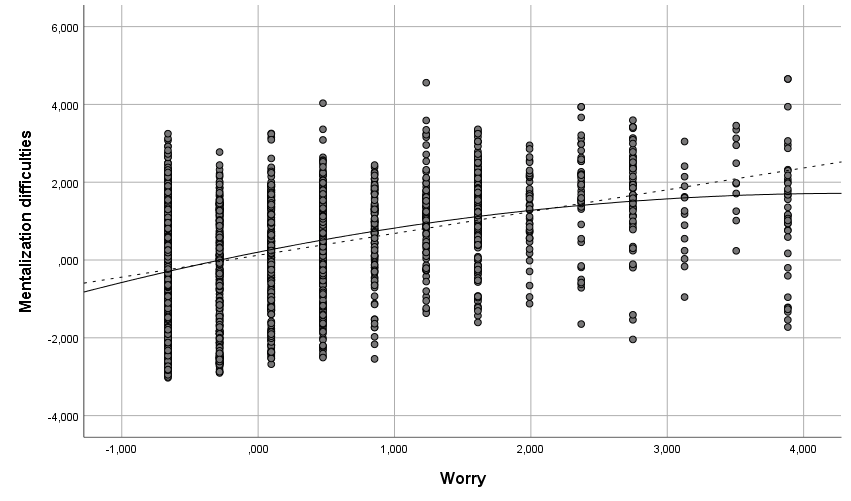


**S4 Figure. Linear and non-linear relationship between worry and mentalization difficulties among young adults. Notes: the solid line shows the quadratic correlation between the variables, and the dashed line shows the linear correlation between the variables.**
