# Supplementary material for: The Reflective Functioning Questionnaire–Revised– 7 (RFQ-R-7): A new measurement model assessing hypomentalization
Source: PLoS One. 2023 Feb 24;18(2):e0282000. doi: 10.1371/journal.pone.0282000 (PMC9956064; doi:10.1371/journal.pone.0282000)
Supplement: S1 Table — (DOCX) [file pone.0282000.s007.docx]

**S1 Table. Factor loadings of the two-factor model by using recoded items**

| Item abbreviation | Item content | **Young adults (*N*=3784)** | | **Adults (*N*=1307)** | |
| --- | --- | --- | --- | --- | --- |
|  |  | **Certainty factor** | **Uncertainty factor** | **Certainty factor** | **Uncertainty factor** |
| Factor loadings | | | | | |
| RFQC1 | People’s thoughts are a mystery to me | 0.62*** | - | 0.45*** | - |
| RFQC2 | I don’t always know why I do what I do | 0.88*** | - | 0.82*** | - |
| RFQC3 | When I get angry I say things without really knowing why I am saying them | 0.91*** | - | 0.88*** | - |
| RFQC4 | When I get angry I say things that I later regret | 0.88*** | - | 0.84*** | - |
| RFQC5 | If I feel insecure I can behave in ways that put others’ backs up | 0.90*** | - | 0.90*** | - |
| RFQC6 | Sometimes I do things without really knowing why | 0.87*** | - | 0.90*** | - |
| RFQU2 | I don’t always know why I do what I do | - | 0.73*** | - | 0.70*** |
| RFQU4 | When I get angry I say things that I later regret | - | 0.71*** | - | 0.71*** |
| RFQU5 | If I feel insecure I can behave in ways that put others’ backs up | - | 0.79*** | - | 0.83*** |
| RFQU6 | Sometimes I do things without really knowing why | - | 0.81*** | - | 0.89*** |
| RFQU7 | I always know what I feel | - | -0.22*** | - | -0.07 |
| RFQU8 | Strong feelings often cloud my thinking | - | 0.67*** | - | 0.70*** |
| Correlation between the two factors | | -0.97*** | | -0.94*** | |

Note. Values related to the items are standardized factor loadings (*λ*). Level of significance: **p*<0.050; ***p*<0.010; ****p*<0.001.
