# Supplementary material for: The Reflective Functioning Questionnaire–Revised– 7 (RFQ-R-7): A new measurement model assessing hypomentalization
Source: PLoS One. 2023 Feb 24;18(2):e0282000. doi: 10.1371/journal.pone.0282000 (PMC9956064; doi:10.1371/journal.pone.0282000)
Supplement: S2 Table — (DOCX) [file pone.0282000.s008.docx]

**S2 Table. Factor loadings of the two-factor model by using the original response scales**

| **Young adults (*N*=3784)** | | | | |
| --- | --- | --- | --- | --- |
|  | **Model with 8 items (RFQ-8)** | | **Model without Item 7**  **(RFQ-R-7)** | |
|  | **Certainty factor** | **Uncertainty factor** | **Certainty factor** | **Uncertainty factor** |
| Factor loadings | | | | |
| People’s thoughts are a mystery to me | 0.52*** | - | 0.52*** | - |
| I don’t always know why I do what I do | 0.37*** | 0.47*** | 0.37*** | 0.47*** |
| When I get angry I say things without really knowing why I am saying them | 0.90*** | - | 0.90*** | - |
| When I get angry I say things that I later regret | 1.10*** | -0.25*** | 1.10*** | -0.25*** |
| If I feel insecure I can behave in ways that put others’ backs up | 0.40*** | 0.47*** | 0.40*** | 0.47*** |
| Sometimes I do things without really knowing why | -0.78*** | 1.66*** | -0.79*** | 1.67*** |
| I always know what I feel (reversed) | - | 0.08*** | Not included | |
| Strong feelings often cloud my thinking | - | 0.77*** | - | 0.77*** |
| Correlation between the two factors | 0.95*** | | 0.95*** | |
| **Adults (*N*=1307)^1^** | | | | |
|  | **Model with 8 items (RFQ-8)** | | **Model without Item 7**  **(RFQ-R-7)** | |
|  | **Certainty factor** | **Uncertainty factor** | **Certainty factor** | **Uncertainty factor** |
| Factor loadings | | | | |
| People’s thoughts are a mystery to me | 0.36*** | - | 0.37*** | - |
| I don’t always know why I do what I do | 0.13* | 0.63*** | 0.10 | 0.65*** |
| When I get angry I say things without really knowing why I am saying them | 0.91*** | - | 0.91*** | - |
| When I get angry I say things that I later regret | 0.94*** | -0.12 | 0.96*** | -0.13 |
| If I feel insecure I can behave in ways that put others’ backs up | 0.27*** | 0.60*** | 0.23*** | 0.64*** |
| Sometimes I do things without really knowing why | -0.58*** | 1.49*** | -0.78*** | 1.68*** |
| I always know what I feel (reversed) | - | 0.22*** | Not included | |
| Strong feelings often cloud my thinking | - | 0.76*** | - | 0.76*** |
| Correlation between the two factors | 0.90*** | | 0.92*** | |

Note. Values related to the items are standardized factor loadings (*λ*). RFQ-8: Brief, 8-item Reflective Functioning Questionnaire. RFQ-R-7: Revised, 7-item Reflective Functioning Questionnaire. Level of significance: **p*<0.050; ***p*<0.010; ****p*<0.001. ^1^The latent variable covariance matrix is not positive definite for both models (due to a negative item residual variance).
