# Supplementary material for: The Reflective Functioning Questionnaire–Revised– 7 (RFQ-R-7): A new measurement model assessing hypomentalization
Source: PLoS One. 2023 Feb 24;18(2):e0282000. doi: 10.1371/journal.pone.0282000 (PMC9956064; doi:10.1371/journal.pone.0282000)
Supplement: S1 Appendix — (DOCX) [file pone.0282000.s009.docx]

**The Reflective Functioning Questionnaire – Revised – 7 (RFQ-R-7)**

Please work through the next 7 statements. For each statement, choose a number between 1 (“Strongly disagree”) and 7 (“Strongly agree”) to say how much you disagree or agree with the statement, and write it beside the statement. Do not think too much about it – your initial responses are usually the best.

|  | **Strongly disagree** |  |  |  |  |  | **Strongly agree** |
| --- | --- | --- | --- | --- | --- | --- | --- |
| 1. People’s thoughts are a mystery to me | 1 | 2 | 3 | 4 | 5 | 6 | 7 |
| 2. I don’t always know why I do what I do | 1 | 2 | 3 | 4 | 5 | 6 | 7 |
| 3. When I get angry I say things without really knowing why I am saying them | 1 | 2 | 3 | 4 | 5 | 6 | 7 |
| 4. When I get angry I say things that I later regret | 1 | 2 | 3 | 4 | 5 | 6 | 7 |
| 5. If I feel insecure I can behave in ways that put others’ backs up | 1 | 2 | 3 | 4 | 5 | 6 | 7 |
| 6. Sometimes I do things without really knowing why | 1 | 2 | 3 | 4 | 5 | 6 | 7 |
| 7. Strong feelings often cloud my thinking | 1 | 2 | 3 | 4 | 5 | 6 | 7 |

Scoring: A total scale score can be calculated by adding up the scores on each item (range of scores: 7-49 points).
